# Introduction to Nanomedicine

**DOI:** 10.3390/molecules21010004

**Published:** 2015-12-22

**Authors:** Didier Astruc

**Affiliations:** ISM, University of Bordeaux, 351 Cours de la Libération, 33405 Talence Cedex, France; didier.astruc@u-bordeaux.fr; Tel.: +33-5-4000-6271; Fax: +33-5-4000-6994

Although mentions of nanoparticles in relation to biomedicine appeared in the late 1970s and are now the subject of over 10,000 publications per year, the term “Nanomedicine” only appeared at the turn of this century, and less than 30 papers including this term were published up to 2005. Ten years later, Web of Science indicates the publication of more than 1000 Nanomedicine articles in 2015 among more than ten times more articles involving nanoparticles for biomedical usage. Nanomedicine has been defined by the European Science Fundation’s forward Look Nanomedicine as follows: “*Nanomedicine uses nano-sized tools for the diagnosis, prevention and treatment of disease and to gain increased understanding of the complex underlying patho-physiology of disease. The ultimate goal is to improve quality of life*.” [1]. It involves the three nanotechnology areas of diagnosis, imaging agents and drug delivery with nanoparticles in the 1–1000 nm range, bioships (from both “top-down” and “bottom-up” sources) and polymer therapeutics [2,3]. A relevant more recent terminology is that of “theranostics” [4,5] involving both diagnostics and therapy with the same nanopharmaceutics.

In fact, Nanomedicine can be traced back to the use of colloidal gold in ancient times [6,7], but Metchnikov and Ehrlich (Nobel Prize for Medicine in 1908) are the modern pioneers of nanomedicine for their works on phagocytosis [8] resp. cell-specific diagnostic and therapy [9]. Seminal works on nanoparticles for nanomedicine were increasingly developed in the last 30 years of the 20th century and included liposomes [10,11], DNA-drug complexes [12], polymer-drug conjugates [13], antibody-drug conjugates [14], polymer nanocapsules [15,16,17], polymer-protein conjugates [18], albumin-drug conjugates [19], block-copolymer micelles [20], anti-arthritis gold nanoparticles [21] and anti-microbial silver nanoparticles [22]. These nanomedicines have various size ranges that are often not strictly within the standard definition of the nanoworld that is 1–100 nm [23]. Clinical toxicities including side effects have been broadly studied and sometimes point toward patient individualization.

Problems that need be overcome are that most drugs are neither specific nor water-soluble. The above nanocarriers have been designed to first solubilize drugs in aqueous media, then serve as nanovectors toward specific targets and control drug release. A majority of nanocarriers used now allow oral drug delivery. Although these nanovectors are designed to translocate across the gastro-intestinal tract, lung, and blood-brain barriers, the amount of drug transferred to the organ is lower than 1%, therefore improvements are challenging [24,25]. Nanovector-drug assemblies are designed to maximize the benefit/risk ratio, and their toxicity must be evaluated not only by sufficiently long term *in vitro* and *in vivo* studies, but also pass multiple clinical studies. For biological assays, these nanomaterials must be characterized very strictly in a fully reproducible way [26,27]. Suitable nanocarriers (including metabolites) must be subjected to research of their antigenicity, immunotoxicity and possible activation of complements (that are a group of serum proteins that activates inflammation, destroys cells and participates in opsonization), pharmacokinetics, biodistribution, and drug release rates [28].

Tumor targeting drugs are a major focus in this context, and they use liposomes, polymers, micelles, conjugates, nanoparticles and conjugates of these nanopharmaceutics [29]. Two main routes are passive targeting using the enhanced permeation and retention (EPR) effect [30,31] and active targeting involving covalent drug attachment using linkers to a receptor that should be specifically recognized by the cancer cells [32]. Drug release rates and stability until the targeted cells are reached are key factors. Imaging using gamma cameras, magnetic resonance (MRI), position emission tomography (PET) and near infrared (NIR) luminescence and fluorescence are major techniques allowing one to quantize drugs in biological fluids and tissues. Active targeting using drug attachment to a receptor is a powerful concept that has been probed for several decades, but progress remains very slow, and positive *in vitro* results are only too rarely confirmed *in vivo*. For instance antibody-targeted radiotherapy was shown to localize less than 0.01% of the administrated dose to the tumor [32]. Evaluation of dose-dependent targeting is essential for pharmacological evaluation, and receptor saturation often occurs at low dose. Biomarkers are required in various nanomedicine technologies to measure the efficacy and safety of these drugs, because only a few % of drugs entering clinical investigation reach marketing approval [33]. Several families of new nanomaterials have attracted increased attention as nanovectors and theranostics in nanomedicine, in particular during the last decade:

- Carbon materials that include fullerene (mainly C_60_), single-wall and multi-wall carbon nanotubes (SWCNTs and MWCTs respectively) [34], graphene oxide (GO) and nanodiamond (ND) [35]. Although these materials are insoluble in most solvents, including aqueous media, they can be polyfunctionalized with solubilizing groups such as polyethylene glycol, *etc.* The carbon cores of the functionalized carbon materials are essentially used as a scaffold, and tumor targeting and imaging using Raman signatures have potential. Although the problem of safety concerning these cores must be addressed, the functional groups ensure protection and penetration into organs. Long-term toxicity remains an issue, however, and clinical tests should be crucial.
- Gold nanoparticles have a many centuries of historic tradition in therapeutics, but nanosciences has brought about novel theranostic concepts based on the medium-sensitive plasmonic absorption resulting from the visible and infrared light-induced collective oscillation of the surface electrons when the nanoparticle size is much smaller than the light wavelength [36,37]. Gold nanoparticle plasmons can be applied in various ways to nanomedicine [38,39,40], in particular photothermal therapy with gold nanorods and hollow gold nanoshells with plasmon bands in the near infrared region and various imaging techniques [37,40]. Gold nanoparticles indeed provide versatile scaffolds for cell surface sensing with the use of both specific recognition and array-based “chemical nose” approaches [41,42,43]. Passive tumor targeting with PEG for EPR effect and active targeting upon covalent linking to rhTNFa (CYT-6091) have reached anticancer clinical trials [44]. The preparation of gold nanoparticles and their functionalization are well controlled and reproducible, which is important for patenting, and the small size of these particles (<10 nm) represents an advantage compared with other nanoparticles that are probed for nanomedicine [36,45]. Although safety studies *in vitro* and *in vivo* are often contradictory, gold nanoparticles are considered as a standard for safety issues [46,47]. Silver and copper nanoparticles also present plasmonic properties, but the gold nanotechnology appears much superior to those of the lighter the group 11 elements. Nethertheless, “nanocrystalline silver” is well known for its established antimicrobial properties [48], although it is also cytotoxic [49].
- Super Paramagnetic Iron Oxide Nanoparticles (SPIONs), usually magnetite, Fe_3_O_4_, are widely explored [50], despite their toxicity [51], in combination with a magnet for magnetic resonance imaging (MRI) and tumor ablation by hyperthermia. This technique has reached clinical use and phase II investigation in brain cancer (multiform glioblastoma) and also clinical study of non-metastatic prostate cancer [52]. Other oxide nanoparticles include silica (usually mesostructured silica) that is used to encapsulate drugs or SPIONs [53,54].
- Quantum Dots (QD), binary semiconductor nanoparticles, are most often CdSe particles coated with ZnS or CdS. They are 2–10 nm dimension fluorescent imaging labels that are frequently used in nanomedicine [55,56] in spite of the toxicity of heavy metals [57].
- Polymers and other macromolecules including co-polymers, antibodies, proteins, aptamers and dendrimers are intensively studied as drug nanovectors in nanomedicine [58,59,60,61,62]. A number of successful polymers are biodegradable and used in pre-clinical and clinical studies [63]. Major advances have been published, but important obstacles still remain concerning the use of encapsulated drugs in polymer nanoparticles including “burst release”, poor drug loading, and poor miscibility of some drugs with the polymer carrier [64]. Dendrimers that are cauliflower-shaped nano-scale macromolecules bearing many functional branch termini [65,66] have considerable capacity to encapsulate drugs and traverse biological barriers [67,68,69,70,71]. The dendritic microbiocide Vivagel was evaluated clinically [72]. Other commercial dendrimers [73] include Ocuseal, a microbial barrier [74], gadomer-17, a dendritic MRI [75], Stratus CS, a cardiac biomarker [76], Alert Ticket for anthrax detection, and Qiagen for *in vitro* DNA transfection [77]. Clinical trials are slow, however. Challenging problems remaining are purity, reproducibility, biodegradability and biocompatibility [78].
- Various forms of liposomes have long been and remain among the most successful drug careers [79]. They include lipids, proteins, albumin, vesicles and related biopolymers and can involve combined drugs such as anti-cancer agents. Combination of imaging agents for diagnostics and drugs for therapy are examples called theranostics.

Many reviews cited in this introduction discuss the various clinical trials of these nano-drugs. Research in nanomedicine is exploding, but multi-phase clinical trials are very demanding. In the end, only a few nanodrug candidates successfully pass regulatory authority requirements. No doubt that interdisciplinary collaborations between biomedical scientists, chemists and biophysicists will in the future favor the arrival of more nanoengineered drugs on the market [80,81,82,83,84].
